# Mild NaCl Stress Influences Staphylococcal Enterotoxin C Transcription in a Time-Dependent Manner and Reduces Protein Expression

**DOI:** 10.3389/fmicb.2022.820067

**Published:** 2022-04-18

**Authors:** Danai Etter, Christina Ukowitz, Corinne Eicher, Taurai Tasara, Sophia Johler

**Affiliations:** ^1^Institute for Food Safety and Hygiene, Vetsuisse Faculty University of Zurich, Zurich, Switzerland; ^2^Laboratory of Food Microbiology, Institute for Food Nutrition and Health, ETH Zurich, Zurich, Switzerland

**Keywords:** stress response, superantigen, food intoxication, virulence gene regulation, SEC variants

## Abstract

Enterotoxins (SEs) produced by *Staphylococcus aureus* are the cause of serious food intoxications. Staphylococcal enterotoxin C (SEC) is one of the main contributors, as it is often highly expressed. *S. aureus* possesses a competitive growth advantage over accompanying bacterial flora under stress conditions encountered in foods, such as high NaCl concentrations. However, the influence of NaCl as an external stressor on SEC expression is still unclear. We investigated the influence of 4.5% NaCl on *sec* mRNA and SEC protein levels. A qRT-PCR assay revealed that NaCl stress leads to time-dependently decreased or elevated *sec* mRNA levels for most strains. SEC protein levels were generally decreased under NaCl stress. Our findings suggest that NaCl stress lowers overall SEC concentration and time-dependently affects *sec* mRNA levels.

## Introduction

*Staphylococcus aureus* is one of the most common causes of foodborne intoxications. In the EU 2454 cases of Staphylococcal Food Poisoning (SFP), outbreaks were reported in 2015 (EFSA, 2016) and 1,400 cases in 2019 (EFSA, 2021). In the USA, the yearly number of cases is estimated at 241′148 (Scallan et al., 2011). Foods commonly implicated in SFP are meat and meat products, as well as dairy products (EFSA, 2017). SFP is caused by staphylococcal enterotoxins (SEs) that are produced by *S. aureus* directly in the food matrix during growth. These exotoxins are of particular interest due to their extreme resistance toward environmental stress conditions, such as heat, acidity, or irradiation. Thus, even heated foods, in which the organism itself has been inactivated, can lead to severe intoxications, as staphylococcal enterotoxins remain emetically and biologically active (Le Loir et al., 2003). To date, a broad range of SEs has been characterized, comprising the classical SEA-SEE and the newly described SEG-SE*l*Z (Fetsch and Johler, 2018). The investigation of staphylococcal enterotoxin C (SEC) is of particular relevance since this toxin can be produced in up to 10 times higher amounts than other SEs (Spaulding et al., 2013). In addition, SEC exists in different, often host-specific variants (Etter et al., 2020). This adds further complexity to the already highly complex interconnected regulatory system of SEs.

Hurdle technologies represent a combination of preservation methods. It is a highly effective tool in the inhibition of growth and toxin formation of pathogenic microorganisms (Leistner, 2000). This includes the addition of preservatives, changing pH, lowering the a_w_ value, and heating or cooling (Rahman, 2020). A common preservation method in foods is the addition of salt. Meat products that are commonly associated with SFP usually have NaCl concentrations ranging from 1–2% for ham or sausages, 2–4.5% for dry fermented sausages, and 5–10% for dried and cured meat (Lilic et al., 2015; Kim et al., 2021). Growth of *S. aureus* is usually inhibited by the surrounding microflora in most food matrices. However, in food matrices with such high salt concentrations, *S. aureus* possesses a remarkable growth advantage over competing bacteria. In fact, *S. aureus* was shown to grow at normal levels in media with up to 20% NaCl (Measures, 1975). While high NaCl concentrations do not effectively impede growth of *S. aureus* in foods, they might still act as a hurdle by influencing SE production. Thus, the presence and even growth of *S. aureus* in foods high in NaCl might not be dangerous to the consumer if enterotoxin formation is sufficiently inhibited. Deciphering the role of NaCl in enterotoxin formation is key to understand the risk related to the presence of *S. aureus* in various foods and could contribute to a reduction in food waste. The WHO aim of reducing sodium content in foods is certainly reasonable regarding dietary requirements (WHO, 2011). Diet-related non-communicable diseases are now the leading cause of mortality worldwide, accounting for more deaths than all other, non-diet-related mortality causes combined (Forouzanfar et al., 2016). Salt reduction may however entail food safety concerns if it facilitates pathogen activity, such as SE production (Doyle and Glass, 2010).

Salt stress acts on bacteria *via* two mechanisms: osmotic effects and specific ion effects (Oren, 1999; Chhabra, 2017). Some of the known stress adaptation mechanisms include accumulation of potassium, amino acids, or sugars to prevent sodium influx and reduce osmotic pressure (Doyle and Glass, 2010). How NaCl stress affects SE expression and regulation has not yet been elucidated. Previous experiments demonstrated a reduction in *sec* under 1.2 M NaCl (*ca*. 7% NaCl) in two strains (Regassa and Betley, 1993). In an older study on the effect of NaCl and pH on SEC protein production, it was not possible to detect SEC in broths with 12% NaCl (Genigeorgis et al., 1971). However, SEC expression has not yet been quantified for different toxin variants and strains from different isolation sources in more realistic circumstances with lower salt concentrations, mimicking those encountered during food production and preservation.

We investigated the influence of 4.5% NaCl stress compared to a low-salt containing control medium on *sec* expression in seven strains from different origins and with different SEC variants and *sec* gene promoters (promoter variants are labeled v1–v4). Potential influence of toxin variants or strain background was assessed on mRNA and protein levels. In addition, quantification on both mRNA and protein levels enabled us to determine whether gene regulation as a NaCl stress response is transcriptional or post-transcriptional. We used 4.5% NaCl containing medium to represent concentrations found in fermented meat products. Our findings contribute to a better understanding of matrix–pathogen interaction that is needed to adapt food production parameters and protect consumer health.

## Materials and Methods

### Bacterial Strains, Growth Conditions, and Sample Collection for *sec* mRNA and SEC Protein Quantification

All *S. aureus* strains and their respective SEC variants in this study are listed in Table 1. The strains were grown in LB medium (BD, Pont-de-Claix, France) (non-stress control conditions, 1% NaCl) and in LB supplemented with a total of 4.5% (w/v) NaCl (BD) (0.77 M). Mild salt stress conditions encountered in food were mimicked by adjusting to a salt concentration typical for dried fermented meat. All media were autoclaved before adding stress conditions and then sterile filtered (Bottle-top filter 0.2 μm, Fisher Scientific, Reinach, Switzerland) and stored at 4°C.

**Table 1:** Overview of *Staphylococcus. aureus* strains used in this study including their SEC variants, origin, and clonal complex.

Strain	Protein variant	*sec* promoter variant	Origin	Clonal complex	References
BW10	SEC^2^	sec_p_ v1	SFP	CC45	1
NB6	SEC^2^	sec_p_ v1	SFP	CC45	2
SAI3	SEC^1^	sec_p_ v3 (H-EMRSA-15)	Human infection	CC8	Wattinger et al., 2012
SAI48	SEC^2^	sec_p_ v1 (79_S10)	Human infection	CC5	Wattinger et al., 2012
SAR1	SEC_bovine_	sec_p_ v2	Bovine mastitis milk	CC151	Johler et al., 2011
SAR38	SEC_bovine_	sec_p_ v2	Bovine mastitis milk	CC151	Johler et al., 2011
OV20	SEC_ovine_	sec_p_ v4	Ovine	CC133	Guinane et al., 2010

1 *Medical Department of the German Federal Armed Forces, Germany*.

2 *Bavarian State Office of Health and Food Safety, Germany*.

Bacteria were grown and sampled according to procedures previously described in Etter et al., 2021. Briefly, colonies from 5% sheep blood agar were cultured in LB broth (16 h at 37°C and 125 rpm). After washing the cultures in 0.85% NaCl suspension, 50 ml of medium (LB and LB + NaCl) were inoculated (OD_600_ = 0.05). Cultures were incubated at 37°C at 125 rpm and sampled after 4 h (exponential phase), 10 h (early stationary phase), and 24 h (late stationary phase). Three independent biological replicates were collected. RNAprotect®Tissue Reagent (Qiagen, Hilden, Germany) was used for mRNA sample stabilization. Low protein binding micro-centrifuge tubes (Thermo Scientific, Waltham, MA USA) were used for protein sample collection.

Growth curves were evaluated by plating serial dilutions on plate count agar (Oxoid, Pratteln, Switzerland) as previously described (Etter et al., 2021).

### RNA Extraction

RNA extraction was performed with the RNeasy mini Kit Plus (Qiagen) as previously described (Sihto et al., 2014; Etter et al., 2021) and quantified with Quantifluor (Promega, Madison, WI USA). Quality control was performed by the Agilent 2,100 Bioanalyzer (Agilent Technologies, Waldbronn, Germany). Samples were included in the study if they met the inclusion criteria of RNA integrity numbers >6. RNA integrity numbers ranged from 6.3–8.2.

### Reverse Transcription and Quantitative Real-Time PCR

All RNA samples were subjected to reverse transcription and qRT-PCR as previously described (Etter et al., 2021). No reverse transcription controls were included as controls in each qPCR run to ensure absence of DNA. Relative expression of the target gene *sec* was normalized using the housekeeping genes *rho* and *rplD* (Sihto et al., 2014). Ct values were determined using the Lightcycler®Software version 1.1.0.1320 (Roche, Basel, Switzerland). Data were expressed as Δct values. Statistical analysis was performed with RStudio 1.3.1093 and GraphPad Prism 9.2.0. For RNA analysis, a mixed effect linear model was fitted on the fold change, with a full three-way interaction between reference gene, strain, and time effects. Fold change was log_10_-transformed to ensure normal distribution in statistical analysis. To determine whether individual mRNA levels were increased (indicated by a fold change significantly larger than 1 we used lsmeans to perform a one-sided effect test, with Holm–Bonferroni-corrected *p*-values). Protein data were log-transformed to ensure normal distribution for statistical analysis. A two-way ANOVA and post-hoc Tukey’s multiple comparisons were performed. Results were regarded as significant if *p* < 0.05. All relevant information according to MIQE guidelines are provided in Supplementary Table 2.

### Protein Quantification

An enzyme-linked immunosorbent assay (ELISA) was performed as previously described (Etter et al., 2021). The protocol was based on Poli and colleagues (Poli et al., 2002) with some modifications according to (Wallin-Carlquist et al., 2010). Antibodies and reference toxins were obtained from Toxin Technology Inc., Sarasota, FL, USA. ELISA measurements were performed in duplicates. Statistical analysis was performed with RStudio 1.3.1093 and GraphPad Prism 9.2.0. Results were regarded as significant if *p* < 0.05.

## Results and Discussion

We measured relative *sec* mRNA levels and quantified SEC protein levels of seven *S. aureus* strains BW10, NB6, SAI3, SAI48, SAR1, SAR38, and OV20 at exponential (4 h), early stationary (10 h), and late stationary (24 h) phase during growth in LB under NaCl (4.5%) stress. Strains BW10 and NB6 (both SEC_2_, v1) were isolated from food, SAI3 (SEC_1_, v3) and SAI48 (SEC_2_, v1) from human infection cases, and SAR1 & SAR38 (both SEC_bovin_e, v2), and OV20 (SEC_ovine_, v4) were isolated from bovine and ovine mastitis cases, respectively.

### *sec* mRNA Levels Observed Under NaCl Stress Correlates With Toxin Variant in a Time-Dependent Manner

**Figure 1:**
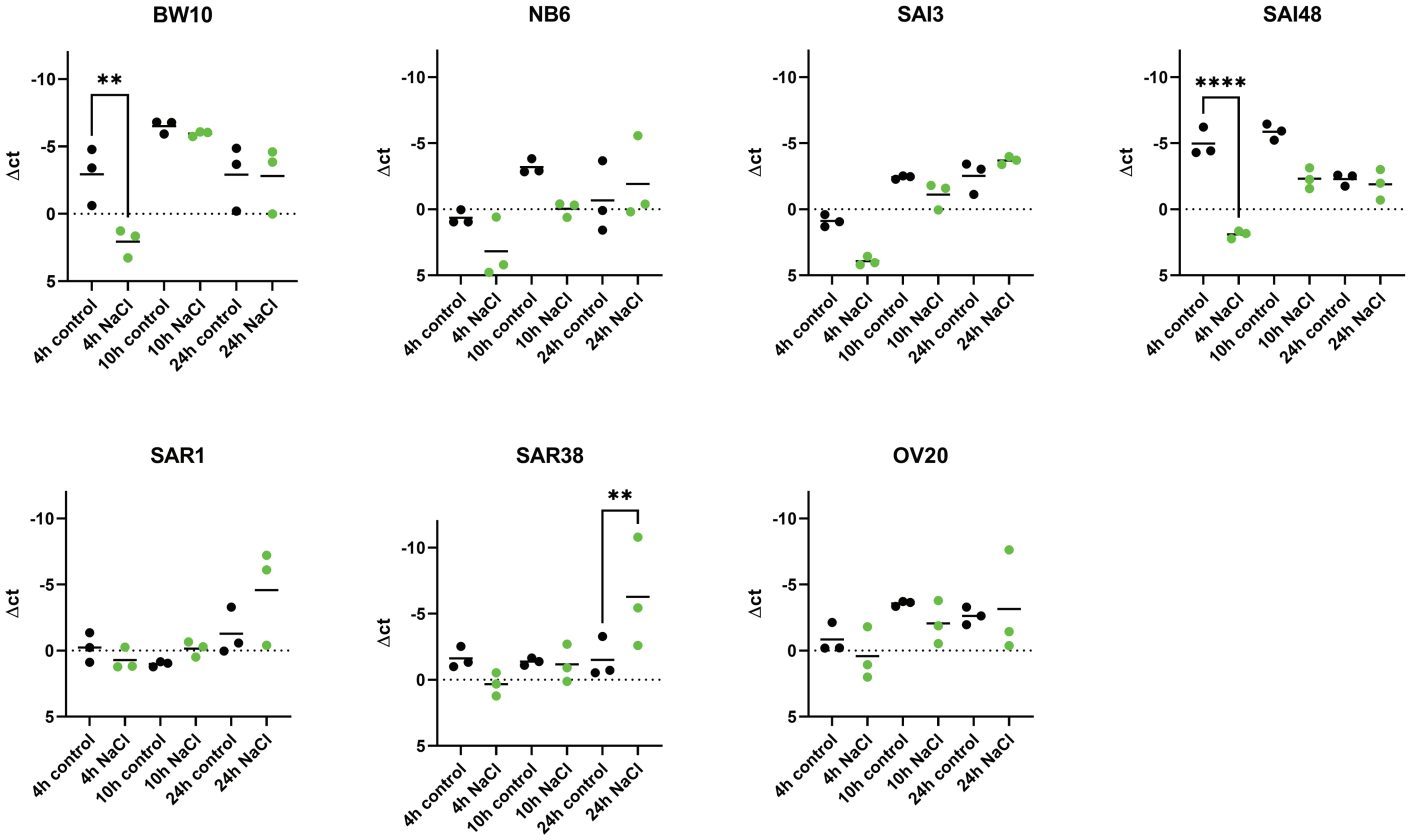
Effect of NaCl stress on *sec* mRNA levels in seven *S. aureus* strains (BW10, NB6, SAI3, SAI48, SAR1, SAR38, OV20) measured by qRT-PCR. qPCR Δct values in exponential (4 h), early stationary (10 h), and late stationary phase (24 h) in LB and LB + 4.5% NaCl for each time point and strain. Control conditions in black and NaCl stress conditions in green. Target mRNA (*sec*) was normalized to two reference genes *rho* and *rplD*. Statistically significant changes in *sec* mRNA levels in LB + 4.5% NaCl compared to LB (*p* < 0.05) are marked by an asterisk.

*sec* mRNA levels were measured in exponential (4 h), early stationary (10 h), and late stationary phase (24 h) across 7 *S. aureus* strains and expressed normalized to strain growth level (Supplementary Figure 1). The expression of *sec* mRNA under NaCl stress tended to be lower compared to control conditions in early growth phases but reached or exceeded control levels in late stationary phase (Figure 1). Six out of seven strains (BW10, NB6, SAI48, SAR1, SAR38, OV20) exhibited significantly altered expression while in one strain (SAI3) *sec* mRNA level was not significantly affected by NaCl stress. Isolates BW10, NB6, and SAI48 who express toxin variant SEC_2_ and promoter variant v1 (Table 1) as well as strains SAR38 and OV20 harboring SEC_bovine_ and promoter variant v3 showed decreased levels of *sec* transcripts in exponential or early stationary phase. Strain SAI3 showed comparable *sec* mRNA level patterns to NB6, although differences observed were not statistically significant. In late stationary phase, all strains either reached (BW10, NB6, SAI3, SAI48, OV20) or surpassed (SAR1, SAR38) control levels. Bovine mastitis isolates SAR1 and SAR38 displayed significantly elevated transcription levels under NaCl stress after 24 h. When the results for the different strains were pooled, there was no significant difference in *sec* expression under NaCl stress at 4 h vs. 10 h. However, at 24 h *sec* transcription was significantly elevated under NaCl stress compared to 10 h as well as 4 h (Supplementary Table 2).

Previous experiments demonstrated a reduction in *sec* mRNA levels under 1.2 M NaCl stress (*ca*. 10% NaCl) in two strains carrying non-specified SEC variants after 24 h incubation (Regassa and Betley, 1993). In contrast, our study found equal or elevated *sec* mRNA levels under mild NaCl stress after 24 h for all seven investigated strains. The level of NaCl stress seems to play a role in transcriptional regulation in the observed strains with higher NaCl concentration leading to more pronounced downregulation. Here, we could show that even a minor increase from 1 to 4.5% NaCl did influence *sec* expression. Therefore, reducing salt in foods could lead to upregulation of *sec* expression and therefore higher SEC concentration in food. The study by Regassa and Betley, 1993, further demonstrated that regulation of SEC occurred at the level of mRNA independently of an intact *agr* allele.

So far, it is not known whether all SEs harbored by a strain react congruently to an external stressor. We included two strains in this study that have previously been tested for transcription of another SE under NaCl stress. Strains BW10 and SAI48 have been investigated in terms of their *sed* transcription under 4.5% NaCl stress. BW10 showed a significant reduction in *sed* mRNA transcription after 24 h, while SAI48 was not affected by NaCl stress (Sihto et al., 2015). In contrast, in the present study *sec* transcription in both strains was significantly reduced in early growth phases but was not affected in late stationary phase. Strains USA300 and HG003 have also been tested for *seb* promoter activity under 4.5% NaCl stress. Promoter activity was significantly downregulated over all time points in both strains (Sihto et al., 2017). In contrast, we observed a downregulation in early growth phases and an upregulation in late stationary phase for *sec*. Comparing the different expression patterns in SEs suggests that each SE can respond differently and that the same stressor can trigger opposing responses in strains that express multiple toxins.

### SEC Protein Reduction Is Strain-Dependent Under NaCl Stress

**Figure 2:**
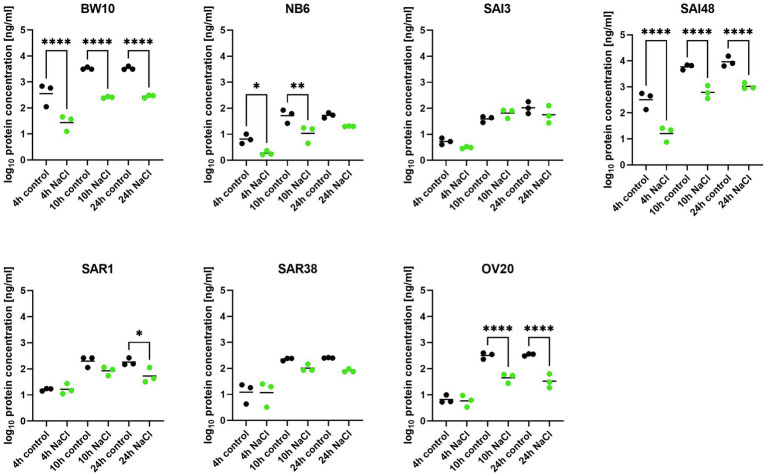
Effect of NaCl stress on SEC protein expression in seven *S. aureus* strains (BW10, NB6, SAI3, SAI48, SAR1, SAR38, OV20) measured by ELISA. Protein concentration is shown in log_10_ values in exponential (4 h), early stationary (10 h), and late stationary phase (24 h) in LB and LB + 4.5% NaCl for each timepoint and strain. Control conditions in black and NaCl stress conditions in green. Statistically significant changes in SEC concentration in LB + 4.5% NaCl compared to LB (*p* < 0.05) are marked by an asterisk.

In addition to *sec* mRNA transcript levels, SEC protein concentrations were also measured by ELISA in exponential (4 h), early stationary (10 h), and late stationary phase (24 h). Strains BW10, NB6, SAI48 (all SEC_2_, promoter v1), SAR1 (SEC_bovine_, promoter v2), and OV20 (SEC_ovine_, promoter v4) produced similar or less SEC under NaCl stress compared to control conditions in some or all growth phases (Figure 2). Reductions ranged from 0 to 1.1 log. For low toxin-producing strains, this observation aligns with a study on the effect of NaCl and pH on SEC protein production where it was not possible to detect SEC in broths with 12% NaCl (Genigeorgis et al., 1971). High toxin producers SAI48 and BW10 were affected by NaCl stress in all growth phases, but still expressed the highest amount of SEC (Table 2). SAI3 and SAR38 were not affected by salt stress, even though SAR38 had reduced and increased *sec* mRNA levels in early exponential and late stationary phase, respectively, under NaCl stress compared to control conditions. With the exception of SAR1 and SAR38, all other strains displayed similar patterns in mRNA and protein expression. These two bovine strains showed an increase in *sec* mRNA but not in protein concentration. Presumably, their SE regulation is fine-tuned differently from the other investigated strains. A previous study demonstrated that osmoregulation of SEC under 1.2 M NaCl stress (*ca*. 7% NaCl) occurred at the level of mRNA (Regassa and Betley, 1993). Our data suggest that SEC regulation under NaCl stress takes place at the mRNA level without additional post-transcriptional regulation under the tested conditions. Even though *sec* transcription was overall upregulated in late stationary phase, a downregulation in early growth phases seems sufficient to decrease final toxin concentration in our strains.

**Table 2:** SEC produced under NaCl stress and control conditions. Absolute values in ng/ml including standard deviation. Effect is shown as difference in percent under NaCl stress compared with non-stress control conditions.

Strains	SEC produced under NaCl stress (ng/ml)																		Effect of NaCl [%]			
	4 h control			10 h control			24 h control			4 h NaCl			10 h NaCl			24 h NaCl			4 h	10 h	24 h	Sum
BW10	462	±	307	3,325	±	360	3,410	±	562	32	±	17	257	±	19	282	±	35	−93	−92	−92	−277
NB6	7	±	3	58	±	29	54	±	12	2	±	0	13	±	7	20	±	1	−72	−78	−63	−213
SAI3	6	±	2	40	±	10	114	±	59	3	±	0	69	±	24	69	±	52	−44	72	−40	−12
SAI48	380	±	220	5,988	±	1,317	9,868	±	4,688	18	±	10	689	±	391	1,076	±	311	−95	−88	−89	−273
SAR1	16	±	2	213	±	87	189	±	65	18	±	9	88	±	31	62	±	44	10	−59	−67	−116
SAR38	15	±	10	228	±	27	254	±	7	16	±	11	106	±	35	82	±	13	5	−53	−68	−116
OV20	7	±	3	328	±	87	345	±	34	6	±	3	47	±	17	38	±	23	−8	−86	−89	−182

A previous study on SEC expression under lactic acid stress showed reductions in SEC protein expressions in a similar range (Etter et al., 2021). Exceptions were observed for strains SAI3 (SEC_1_, promoter v3) and SAI48 (SEC_2_, promoter v1). SAI3 was only marginally affected by NaCl stress with an overall reduction of −21%, but lactic acid stress had led to a pronounced depletion of −109%. SAI48 on the other hand was significantly affected by NaCl stress with −273% SEC expression reduction, but only showed −29% reduction under lactic acid stress (Table 2). Likely, different stress response pathways trigger alternate regulatory elements depending on the genetic background of the strain.

## Conclusion and Outlook

*Staphylococcus. aureus* is commonly present in many foods. Whether presence and growth of this organism is harmful to the consumer heavily depends on its ability to form SEs. It has been shown that food matrix components can influence toxin expression (Alibayov et al., 2015; Sihto et al., 2015; Susilo et al., 2017). We demonstrated here that an increase in NaCl can decrease *sec* transcription mainly at the beginning of growth for many strains. Although, some strains showed elevated transcription levels in late stationary phase. Intriguingly, all strains (BW10, NB6, SAI48) carrying gene variant SEC_2_/promoter v1 downregulated *sec* transcription in exponential or early stationary phase. Strains harboring SEC_bovine_/promoter v2 (SAR1, SAR38) did upregulate *sec* transcription after 24 h. No influence of strain origin or clonal complex was apparent. Due to the limited number of strains used trends related to strain origin or genetic background might not be detectable. SEC protein expression was overall lowered under NaCl stress conditions for all strains. Strains SAI3 and SAI48 showed a different behavior when exposed to NaCl stress than to lactic acid stress. These applied stressors likely trigger different stress response pathways. Different behaviors in *sec* and *sed* expression under NaCl stress might arise from different encoding regions of both SEs. *sec* is encoded on a Staphylococcal pathogenicity island (SaPI), while *sed* is usually encoded on a plasmid. Our results show that findings on transcriptional regulation must not be extrapolated from one SE to another. The different behaviors highlight the importance of investigating a diverse strain set when it comes to virulence factor regulation in *S. aureus*. While transcriptional data does not provide reliable information on the final toxin concentration in foods, it is important to uncover transcriptional regulatory processes. Only by combining transcriptional data and protein expression can a clear picture of stress response and regulatory mechanisms as well as toxic potential be drawn. The investigation of transcriptional and protein expression enabled us to confirm that *sec* regulation under NaCl stress occurs at mRNA level without relevant post-transcriptional modifications in our strain set. Further studies are needed to clarify, which regulatory elements are triggered by the respective stressors. Sequencing of regulatory elements and transcriptomic studies could shed light on the complex interplay of regulators. Our study shows that NaCl could contribute as a hurdle to lower SE concentration in foods containing NaCl. In regard to food, it can be concluded that an increase to 4.5% NaCl leads to lower SEC concentrations for most *S. aureus* strains. Our study highlights the importance of matrix–pathogen interactions in foods and contributes toward a more conscious formulation of food products.

## Data Availability Statement

The original contributions presented in the study are included in the article/Supplementary Material, further inquiries can be directed to the corresponding author.

## Author Contributions

SJ, TT, and DE contributed conception and design of the study and wrote sections of the manuscript. DE, CU, and CE analyzed the data and were responsible for data acquisition. DE wrote the first draft of the manuscript. All authors contributed to manuscript revision, read and approved the submitted version.

## Conflict of Interest

The authors declare that the research was conducted in the absence of any commercial or financial relationships that could be construed as a potential conflict of interest.

## Publisher’s Note

All claims expressed in this article are solely those of the authors and do not necessarily represent those of their affiliated organizations, or those of the publisher, the editors and the reviewers. Any product that may be evaluated in this article, or claim that may be made by its manufacturer, is not guaranteed or endorsed by the publisher.
