# Supplementary material for: Mild NaCl Stress Influences Staphylococcal Enterotoxin C Transcription in a Time-Dependent Manner and Reduces Protein Expression
Source: Front Microbiol. 2022 Apr 18;13:820067. doi: 10.3389/fmicb.2022.820067 (PMC9062481; doi:10.3389/fmicb.2022.820067)
Supplement: Supplementary file 1 [file Data_Sheet_1.docx]

Supplementary Material

**Supplementary Figure S1.** Growth curves under NaCl stress and control conditions. Growth was compared for strains BW10, NB6, SAI3, SAI48, SAR1, SAR38, and OV20. Control conditions are shown in blue, NaCl stress in red. Data points show mean values, error bars depict standard deviations.

**Supplementary Table S2** Comparison of *sec* mRNA expression under NaCl stress compared to control conditions at different time points. Significant differences are marked with asterisks under Summary. One-way ANOVA and Tukey’s multiple comparisons were done in GraphPad Prism 9.2.0.

| Number of families | 1 |  |  |  |  |  |  |  |
| --- | --- | --- | --- | --- | --- | --- | --- | --- |
| Number of comparisons per family | 3 |  |  |  |  |  |  |  |
| Alpha | 0.05 |  |  |  |  |  |  |  |
|  |  |  |  |  |  |  |  |  |
| Tukey's multiple comparisons test | Mean Diff. | 95.00% CI of diff. | Below threshold? | Summary | Adjusted P Value |  |  |  |
| 4h vs. 10h | -0.5070 | -1.289 to 0.2751 | No | ns | 0.2495 | A-B |  |  |
| 4h vs. 24h | -1.544 | -2.326 to -0.7622 | Yes | *** | 0.0002 | A-C |  |  |
| 10h vs. 24h | -1.037 | -1.819 to -0.2552 | Yes | ** | 0.0088 | B-C |  |  |
|  |  |  |  |  |  |  |  |  |
| Test details | Mean 1 | Mean 2 | Mean Diff. | SE of diff. | n1 | n2 | q | DF |
| 4h vs. 10h | -0.8588 | -0.3518 | -0.5070 | 0.3064 | 7 | 7 | 2.340 | 18 |
| 4h vs. 24h | -0.8588 | 0.6855 | -1.544 | 0.3064 | 7 | 7 | 7.127 | 18 |
| 10h vs. 24h | -0.3518 | 0.6855 | -1.037 | 0.3064 | 7 | 7 | 4.787 | 18 |
