# Supplementary material for: Mild NaCl Stress Influences Staphylococcal Enterotoxin C Transcription in a Time-Dependent Manner and Reduces Protein Expression
Source: Front Microbiol. 2022 Apr 18;13:820067. doi: 10.3389/fmicb.2022.820067 (PMC9062481; doi:10.3389/fmicb.2022.820067)
Supplement: Supplementary file 2 [file Table_2.docx]

**MIQE data**

| **ITEM TO CHECK** | **IMPORTANCE** | CHECKLIST |
| --- | --- | --- |
| **EXPERIMENTAL DESIGN** |  |  |
| Definition of experimental and control groups | **E** | NaCl stress vs. control medium |
| Number within each group | **E** | 7 strains at 3 timepoints in 3 replicates = 63 |
| Assay carried out by core lab or investigator's lab? | D | Investigator’s lab |
| Acknowledgement of authors' contributions | D | - |
| **SAMPLE** |  |  |
| Description | **E** | Liquid bacterial culture |
| Volume/mass of sample processed | D | 1 ml |
| Microdissection or macrodissection | **E** | Not applicable |
| Processing procedure | **E** | Not applicable |
| If frozen - how and how quickly? | **E** | - |
| If fixed - with what, how quickly? | **E** | - |
| Sample storage conditions and duration (especially for FFPE samples) | **E** | Storage in RNAprotect solution at -20 °C for max. 7 days |
| **NUCLEIC ACID EXTRACTION** |  |  |
| Procedure and/or instrumentation | **E** | Total RNA extraction |
| Name of kit and details of any modifications | **E** | RNeasy, Qiagen |
| Source of additional reagents used | D | DNase, Qiagen |
| Details of DNase or RNAse treatment | **E** | Enzymatic and column DNase treatment |
| Contamination assessment (DNA or RNA) | **E** | No reverse transcription controls for DNA contamination detection |
| Nucleic acid quantification | **E** | Promega Quantus fluorometer |
| Instrument and method | **E** | Roche LightCycler 96 |
| Purity (A260/A280) | D | - |
| Yield | D | - |
| RNA integrity method/instrument | **E** | Agilent RNA 6000 pico kit |
| RIN/RQI or Cq of 3' and 5' transcripts | **E** | RIN 6.3 or higher |
| Electrophoresis traces | D | - |
| Inhibition testing (Cq dilutions, spike or other) | **E** | Not done |
| **REVERSE TRANSCRIPTION** |  |  |
| Complete reaction conditions | **E** | QuantiTect Reverse Transcription Kit according to manufacturer’s instructions: 2 min 2 μl gDNA wipeout buffer and 12 μl sample at 42 °C, add 1 μl reverse transcriptase + 1 μl RT primer + 4 μl buffer, 15 min at 42 °C, 3 min at 95 °C. |
| Amount of RNA and reaction volume | **E** | 40 ng/μl RNA in 12 μl reaction volume |
| Priming oligonucleotide (if using GSP) and concentration | **E** | Qiagen RT kit uses a mixture of random hexamers and oligos as primers |
| Reverse transcriptase and concentration | **E** | Quantiscript Reverse Transcriptase |
| Temperature and time | **E** | 15 min at 42 °C |
| Manufacturer of reagents and catalogue numbers | D | Qiagen |
| Cqs with and without RT | D* | - |
| Storage conditions of cDNA | D | - |
| **qPCR TARGET INFORMATION** |  |  |
| If multiplex, efficiency and LOD of each assay. | **E** | - |
| Sequence accession number | **E** | KX168612, KX168613, KX168614, KX168615 |
| Location of amplicon | D | - |
| Amplicon length | **E** | 109 |
| *In silico* specificity screen (BLAST, etc) | **E** | BLAST |
| Pseudogenes, retropseudogenes or other homologs? | D | - |
| Sequence alignment | D | - |
| Secondary structure analysis of amplicon | D | - |
| Location of each primer by exon or intron (if applicable) | **E** | Not applicable |
| What splice variants are targeted? | **E** | Not applicable |
| **qPCR OLIGONUCLEOTIDES** |  |  |
| Primer sequences | **E** | TAACGGCAATACTTTTTGGT  AGGTGGACTTCTATCTTCAC |
| RTPrimerDB Identification Number | D | - |
| Probe sequences | D** | - |
| Location and identity of any modifications | **E** | Not applicable |
| Manufacturer of oligonucleotides | D | - |
| Purification method | D | - |
| **qPCR PROTOCOL** |  |  |
| Complete reaction conditions | **E** | \|  \| °C \| acquisition \| time \| ramp rate °C/s \| Acquisitions/°C \| \| --- \| --- \| --- \| --- \| --- \| --- \| \| denaturation \| 95 \| - \| 00:10:00 \| 4.4 \|  \| \| **40x** amplification \| 95 \| - \| 00:00:15 \| 4.4 \|  \| \| 52 \| - \| 00:00:30 \| 2.2 \|  \| \| 72 \| - \| 00:00:30 \| 4.4 \|  \| \| 78 \| single \| 00:00:01 \| 4.4 \|  \| \| melting \| 95 \| - \| 00:00:05 \| 4.4 \|  \| \| 65 \| - \| 00:01:01 \| 2.2 \|  \| \| 97 \| continuous \|  \| 0.11 \| 5 \| \| cooling \| 40 \| - \| 00:00:10 \| 1.5 \|  \| |
| Reaction volume and amount of cDNA/DNA | **E** | 20 μl reaction volume, 0.5 μl of cDNA sample |
| Primer, (probe), Mg++ and dNTP concentrations | **E** | FastStart Essential DNA Green Master |
| Polymerase identity and concentration | **E** | FastStart Taq DNA Polymerase |
| Buffer/kit identity and manufacturer | **E** | FastStart Essential DNA Green Master |
| Exact chemical constitution of the buffer | D | - |
| Additives (SYBR Green I, DMSO, etc.) | **E** | SYBR Green |
| Manufacturer of plates/tubes and catalog number | D | - |
| Complete thermocycling parameters | **E** | See reaction conditions |
| Reaction setup (manual/robotic) | D | - |
| Manufacturer of qPCR instrument | **E** | Roche |
| **qPCR VALIDATION** |  |  |
| Evidence of optimisation (from gradients) | D | - |
| Specificity (gel, sequence, melt, or digest) | **E** | melt |
| For SYBR Green I, Cq of the NTC | **E** | >40 |
| Standard curves with slope and y-intercept | **E** | yes |
| PCR efficiency calculated from slope | **E** | Yes |
| Confidence interval for PCR efficiency or standard error | D | - |
| r2 of standard curve | **E** | 0.9831 |
| Linear dynamic range | **E** | 18-27 Cq |
| Cq variation at lower limit | **E** | not determined |
| Confidence intervals throughout range | D | - |
| Evidence for limit of detection | **E** | not determined |
| If multiplex, efficiency and LOD of each assay. | **E** | - |
| **DATA ANALYSIS** |  |  |
| qPCR analysis program (source, version) | **E** | LightCycler® Software 1.5 |
| Cq method determination | **E** | Second derivative maximum method |
| Outlier identification and disposition | **E** | Based on Cq error range |
| Results of NTCs | **E** | Negative |
| Justification of number and choice of reference genes | **E** | Previous publication about reference gene validation (Sihto et al., 2014). |
| Description of normalisation method | **E** | Reference gene |
| Number and concordance of biological replicates | D | 3 |
| Number and stage (RT or qPCR) of technical replicates | **E** | 2 |
| Repeatability (intra-assay variation) | E | 0.3-0.5 Cp variation |
| Reproducibility (inter-assay variation, %CV) | D | - |
| Power analysis | D | - |
| Statistical methods for result significance | **E** | ANOVA and Tukey’s multiple comparisons |
| Software (source, version) | E | GraphPad Prism 9 |
| Cq or raw data submission using RDML | **D** | - |
